# Dissecting the contribution of Gag domain in resistance development in HIV-1 patients failing ARV therapy

**DOI:** 10.1186/1742-4690-10-S1-P10

**Published:** 2013-09-19

**Authors:** Ilaria Carli, Claudia Del Vecchio, Saverio G Parisi, Federico Dal Bello, Arianna Calistri, Giorgio Palù, Cristina Parolin

**Affiliations:** 1 Department of Molecular Medicine, University of Padua, Padua, Italy; 2 Department of Biology, University of Padua, Padua, Italy

## Background

The Human Immunodeficiency virus type 1 (HIV-1) protease (PR) and reverse transcriptase (RT) are key enzymes in viral replication and major target of Antiretroviral (ARV) therapy. The mechanisms of resistance mainly involve mutations altering the interaction of viral enzymes and inhibitors. Recent studies reveal that, besides the enzymes-encoding ones, other regions might contribute to the development of resistance. In particular, some specific cleavage and non-cleavage site mutations in Gag increase the replication ability of mutant viruses. The effect of amino acid substitutions in different domains of Gag selected *in vivo* on viral replication, remains to be elucidated. In attempt to clarify this aspect, we analyzed clinical samples of HIV-1 infected patients failing PR Inhibitor (PI) and RT Inhibitor (RTI) treatment among a cohort of five infectious diseases units located in Veneto in northeastern Italy.

## Materials and methods

Plasma and PBMCs samples from ARV treatment patients were used for *gag* and *pol* gene PCR amplification and sequencing. Selected patient PCR-derived *gag* products were adopted to reconstitute recombinant HIV-1 viruses in an otherwise wildtype background (HIV-1 LAI). Gag processing experiments, single-cycle infectivity assay and replication kinetics were performed.

## Results

We analyzed the *gag* and *pol* sequences derived from clinical samples of HIV-1 infected patients failing PR Inhibitors (PIs) and RT Inhibitors (RTIs). Besides polymorphisms and multiple amino acid substitutions associated with inhibitor resistance, genotypic analyses have identified insertions within the Gag at the level of the matrix domain and the p6 domains. The effect of these mutations have been examined in terms of: (i) Gag and Gag-Pol processing and particle release; (ii) virus infectivity as well as iii) viral replication capacity.

## Conclusions

Our results contribute to better characterizing the role of Gag and the relationship to PR and RT in resistance development, their relevance in viral replication and evolution in the presence or in the absence of drugs.

